# Microenvironment‐induced PIM kinases promote CXCR4‐triggered mTOR pathway required for chronic lymphocytic leukaemia cell migration

**DOI:** 10.1111/jcmm.13632

**Published:** 2018-04-17

**Authors:** Emilia Białopiotrowicz, Patryk Górniak, Monika Noyszewska‐Kania, Bartosz Puła, Hanna Makuch‐Łasica, Grażyna Nowak, Aleksandra Bluszcz, Maciej Szydłowski, Ewa Jabłonska, Karolina Piechna, Tomasz Sewastianik, Anna Polak, Ewa Lech‐Marańda, Bożena K. Budziszewska, Maja Wasylecka‐Juszczyńska, Katarzyna Borg, Krzysztof Warzocha, Wojciech Czardybon, Michał Gałęzowski, Renata Windak, Krzysztof Brzózka, Przemysław Juszczyński

**Affiliations:** ^1^ Department of Experimental Hematology Institute of Hematology and Transfusion Medicine Warsaw Poland; ^2^ Department of Hematology Institute of Hematology and Transfusion Medicine Warsaw Poland; ^3^ Department of Diagnostic Hematology Institute of Hematology and Transfusion Medicine Warsaw Poland; ^4^ Department of Hematology and Transfusion Medicine Centre of Postgraduate Medical Education Warsaw Poland; ^5^ Selvita S.A. Cracow Poland

**Keywords:** chronic lymphocytic leukaemia, CXCR4, mTOR, PIM kinase

## Abstract

Lymph node microenvironment provides chronic lymphocytic leukaemia (CLL) cells with signals promoting their survival and granting resistance to chemotherapeutics. CLL cells overexpress PIM kinases, which regulate apoptosis, cell cycle and migration. We demonstrate that BCR crosslinking, CD40 stimulation, and coculture with stromal cells increases PIMs expression in CLL cells, indicating microenvironment‐dependent PIMs regulation. PIM1 and PIM2 expression at diagnosis was higher in patients with advanced disease (Binet C vs. Binet A/B) and in those, who progressed after first‐line treatment. In primary CLL cells, inhibition of PIM kinases with a pan‐PIM inhibitor, SEL24‐B489, decreased PIM‐specific substrate phosphorylation and induced dose‐dependent apoptosis in leukaemic, but not in normal B cells. Cytotoxicity of SEL24‐B489 was similar in *TP53*‐mutant and *TP53* wild‐type cells. Finally, inhibition of PIM kinases decreased CXCR4‐mediated cell chemotaxis in two related mechanisms‐by decreasing CXCR4 phosphorylation and surface expression, and by limiting CXCR4‐triggered mTOR pathway activity. Importantly, PIM and mTOR inhibitors similarly impaired migration, indicating that CXCL12‐triggered mTOR is required for CLL cell chemotaxis. Given the microenvironment‐modulated PIM expression, their pro‐survival function and a role of PIMs in CXCR4‐induced migration, inhibition of these kinases might override microenvironmental protection and be an attractive therapeutic strategy in this disease.

## 1 INTRODUCTION

B‐cell chronic lymphocytic leukaemia (CLL) is characterized by progressive accumulation of mature monoclonal B cells in the peripheral blood, bone marrow and secondary lymphoid tissues.^1^ Several prognostic markers, such as the Rai and Binet staging systems, *TP53* and other cytogenetic abnormalities, ZAP70 expression and immunoglobulin heavy variable (*IGHV*) gene mutational status can be used to predict the survival outcome of patients with CLL.^2^ *IGHV* mutations distinguish two main biologically distinct subtypes of the disease, with different underlying genetic lesions, degree of clonal evolution, epigenetic changes and activated signalling pathways. The mutated *IGHV* subtype is associated with a good prognosis and the unmutated *IGHV* subtype with a poor prognosis.^1^

While majority of circulating CLL cells are arrested in the G0 phase of the cell cycle, replenishment of the leukaemic population is dependent on a proliferating fraction in the bone marrow and lymphoid tissues.^3^ In these compartments CLL cells interact with multiple bystander cell types, including bone marrow stromal cells (BMSCs), nurse‐like cells (NLCs), follicular dendritic cells (FDCs), endothelial cells and T cells.^4^ These microenvironment components create niches that communicate with CLL cells *via* direct contact and paracrine signals, protecting them from spontaneous and drug‐induced apoptosis, and fostering proliferation. Consistent with this, primary CLL cells isolated from lymph nodes exhibit gene expression signatures characterized by activation of the B‐cell receptor (BCR) pathway, NFκB pathway and increased expression of E2F target genes.^5^ Trafficking of neoplastic B cells to these proliferation‐conducive compartments is controlled by chemokines.^6^, ^7^ One of the key chemokines involved in CLL cells homing is CXCL12 (formerly stromal‐cell derived factor 1, SDF1). Activation of CXCR4 induces CLL cells chemotaxis, transendothelial migration and exhibits direct anti‐apoptotic effects.^8^, ^9^, ^10^, ^11^ Given the role of CXCR4 in CLL cell motility and viability, mechanisms regulating CXCR4 activity and CXCR4‐triggered signal transduction are particularly interesting as potential therapeutic targets. Accordingly, highly active B‐cell receptor signalling inhibitors, such as ibrutinib, lead to egress of CLL cells from the lymphoid compartments to a periphery in a mechanism that involves decrease of surface CXCR4 expression.^8^

CXCR4 surface expression and recycling are regulated by PIM (provirus integration site for Moloney murine leukaemia virus) kinases, which phosphorylate CXCR4 on serine 339.^9^ PIMs have been postulated as a key mechanism downstream of BCR, responsible for modulation of CXCR4 in CLL.^8^, ^10^ The family of PIM proteins involves three conserved oncogenic serine/threonine kinases, PIM1, PIM2 and PIM3. PIMs phosphorylate a broad range of substrates, which are engaged in cell growth, metabolism, proliferation, migration and drug resistance.^12^, ^13^, ^14^ Increased activity of PIM kinases consolidates multiple oncogenic pathways by phosphorylation and inactivation of Forkhead box O (FOXO) family tumour suppressors, inactivation of proapoptotic Bcl‐2‐associated death promoter (BAD) and MYC stabilization.^15^ Moreover, PIM kinases phosphorylate 4E‐binding protein 1 (4EBP1) and thus promote protein translation and tumour growth.^16^, ^17^, ^18^ Given these pleiotropic effects, inhibition of PIM kinases appeared a highly promising therapeutic strategy in multiple human malignancies, including lymphoma. In this study, we investigated the expression of PIM kinases in CLL patients and further characterized the consequences of their inhibition. We demonstrate that PIMs expression is induced by the microenvironment‐derived signals. Blocking PIMs activity with a newly developed small molecule inhibitor SEL24‐B489 overrides protective microenvironment signals and induces CLL cell death. PIM inhibition blocks CLL cells migration in the CXCL12 chemokine gradient by affecting CXCR4 surface expression and CXCR4‐dependent mTOR activation. Consistent with these pathogenetic findings, we demonstrate that expression of individual PIM isoforms is higher in patients with more aggressive and advanced disease. Thus, PIM kinases directly support CLL cell survival and participate in the cross‐talk between leukaemic cells and their microenvironment.

## 2 METHODS

### 2.1 CLL patient samples and cell culture

The study enrolled 141 newly diagnosed and 9 relapsed CLL patients, and was conducted after local bioethics committee approval and according to Declaration of Helsinki. Patient baseline characteristics are given in Table 1. Peripheral blood mononuclear cells were separated by Ficoll gradient centrifugation. B cells were isolated with the B cell isolation kit II (Miltenyi Biotec). After isolation CLL cells were maintained in RPMI‐1640 medium supplemented with 10% autologous plasma, 10% FBS, 1% penicillin‐streptomycin and 25 mmol/L HEPES buffer, at a density of 1 × 10^7^ cells/mL. For co‐culture experiments CLL cells were layered over the 30%‐confluent HS5 stromal cells and treated as indicated. After 48 hours CLL cells were harvested by gentle agitation and further processed as described.

**Table 1:** Baseline characteristics of CLL patients included in the study

CLL cohort (n = 150)	Frequency (%)	Median (range)
Clinical characteristics		
Age, years		65 (37‐87)
Sex		
Male	95 (63)	
Female	55 (37)	
Newly diagnosed	141 (94)	
Progression during follow up	25 (17)	
Relapsed	9 (6)	
Binet		
A	71 (47)	
B	56 (38)	
C	23 (15)	
Rai		
0	14 (9)	
I‐II	103 (69)	
III‐IV	33 (22)	
WBC (g/L)		49.8 (3.4‐755.5)
HGB (g/dL)		12.7 (4.8‐17.2)
PLT (g/L)		155 (6‐382)
LDH (n = 147)^a^		
>480 U/L	27 (18)	
≤480 U/L	120 (82)	
sB2‐M (n = 119)^a^		
>2.20 mg/L	101 (85)	
≤2.20 mg/L	18 (15)	
IGHV status (n = 108)^a^		
Unmutated (U‐CLL)	66 (61)	
Mutated (M‐CLL)	42 (39)	
FISH (n = 141)^a^		
Del 13q14	89 (63)	
Del 11q23	23 (16)	
Trisomy 12	15 (11)	
Del 17p13	7 (5)	
FISH – negative	30 (21)	

a Number of patients is indicated in parentheses when the entire cohort was not investigated for that variable.

### 2.2 BCR crosslinking and CD40 stimulation

For BCR crosslinking 1 × 10^7^ cells were treated with goat anti‐human IgM F(ab′)2 (Jackson ImmunoResearch) at 10 μg/mL final concentration for 8‐24 hours. CD40 ligand (R&D Systems) was used at 50 ng/mL final concentration for 1 hour.

### 2.3 Quantitative PCR

RNA was isolated using Universal RNA Purification Kit (EURx, Gdansk, Poland) and transcribed to cDNA with Transcriptor First Strand cDNA Synthesis Kit (Roche). Transcript abundance was measured using SYBR Green PCR Master Mix (Applied Biosystems) and LightCycler 480 as described.^19^ In brief, obtained CT values for individual PIM isoforms and housekeeping control (glyceraldehyde‐3‐phosphate dehydrogenase; GAPDH) were used to calculate relative transcript abundance, using the 2^−ΔΔCT^ method.^19^ Primer sequences are given in Table S1.

### 2.4 Chemicals, apoptosis and migration assays

Pan‐PIM kinase inhibitors SEL24‐B489 and AZD1208 were kindly provided by Selvita S.A.^20^ The mTOR inhibitor OSI‐027 was purchased from SelleckChem (Houston, TX, USA). After incubation with the compounds, CLL cells were stained with AnnexinV‐PE/7AAD kit (BD Biosciences) and analysed using the FACS Canto II. Annexin V‐positive and double Annexin V/7AAD‐positive cells were considered apoptotic. Migration assays were performed using transwell system (Corning, NY, USA). Briefly, CLL cells were incubated for 10 hours with 10 μmol/L SEL24‐B489 or vehicle (dimethyl sulfoxide, DMSO); thereafter cells were counted and 5 × 10^6^ cells were placed in the top chamber of the transwell dish (24‐well plate format). The lower chamber contained medium with 500 ng/mL CXCL12 (R&D Systems). After 6 hours incubation (37°C, 5% CO_2_) cells migrated into the lower chamber were counted using trypan blue exclusion assay.

### 2.5 Immunoblotting

Protein extracts were prepared using RIPA buffer as previously described.^21^ Protein extracts were PAGE‐separated, electrotransferred to PVDF membranes (Millipore) and immunoblotted with primary and appropriate secondary antibodies (Table S2). Signals were detected with the Western Lighting ECL (Perkin Elmer, Waltham, MA, USA) using G:BOX Chemi XT4 (Syngene, Frederick, MD, USA). Densitometric quantifications of band intensities were performed using Image Studio Lite software (https://www.licor.com/bio/products/software/image_studio_lite/). PIM1/PIM2 levels were quantified relative to a pooled sample from all investigated patients, mixed at equal amounts and assigned as an arbitrary value 1. Quantified GAPDH was used as an internal control to normalize protein loading between samples.

### 2.6 CXCR4 surface expression

Chronic lymphocytic leukaemia cells were incubated for 2‐10 hours with 10 μmol/L SEL24‐B489 or vehicle (DMSO), washed and stained with APC‐conjugated CXCR4 antibody or APC‐conjugated mouse IgG2a, κ as an isotype control (Table S2), and analysed using the FACS Canto II.

### 2.7 Statistical analysis

Comparisons between variables were performed with GraphPad Prism 6 software (GraphPad, La Jolla, CA, USA), using indicated tests; *P* < .05 was considered statistically significant.

## 3 RESULTS

### 3.1 PIM1 and PIM2 are associated with unfavourable CLL prognosis

**Figure 1:**
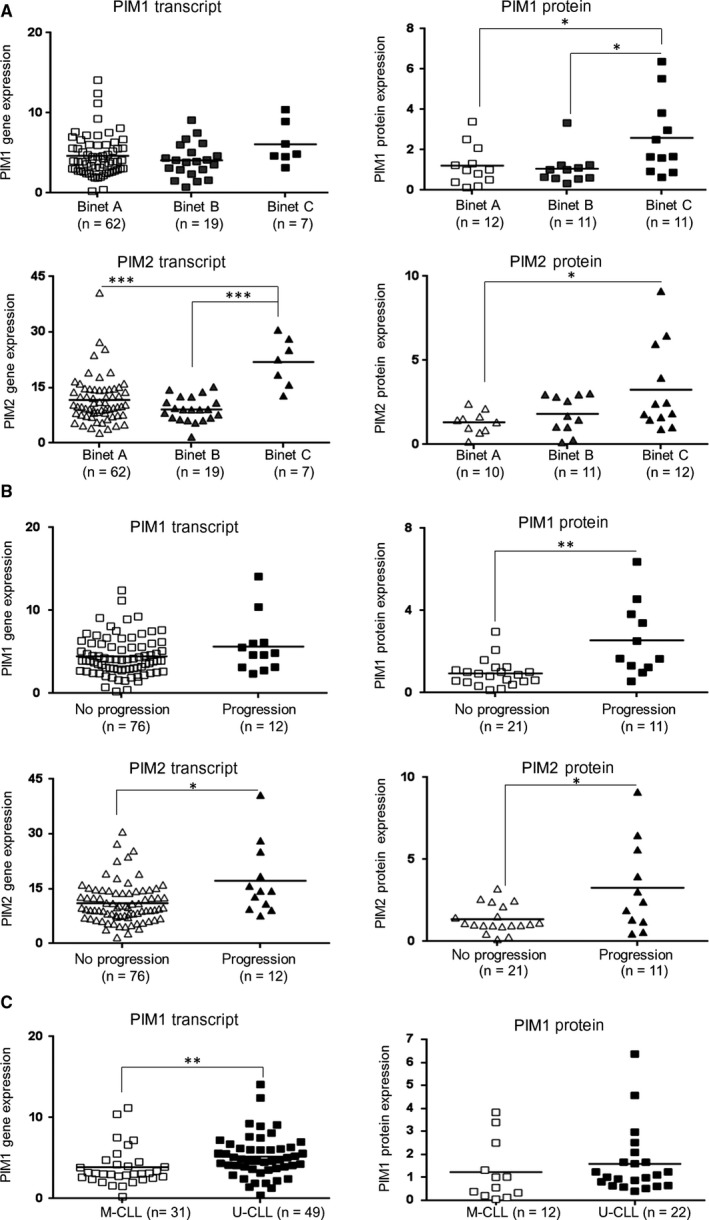
PIM expression is associated with CLL clinical parameters. PIM1/2 transcript levels were assessed by qPCR in 88 newly diagnosed CLL patients. Relative abundance of PIM1/2 transcripts was determined using 2^−ΔΔ^ ^CT^ method, with *GAPDH* used as a reference gene. PIM1/2 protein expression was determined by densitometric quantification of Western blots. GAPDH protein was used as a loading control. (A) PIM1 protein and PIM2 transcript/protein levels are significantly higher in patients with advanced CLL (Binet C), compared to subjects in earlier disease stages (Binet A/B). Please see Supplemental Figure 2A for example source Western blots. (B) PIM1 protein and PIM2 transcript/protein levels (at diagnosis) are higher in patients who eventually progressed after first‐line treatment. Please see Supplemental Figure 2B for example source Western blots. (C) PIM1 transcript abundance is significantly elevated in patients with unmutated *IGHV* status (U‐CLL) comparing to subjects with mutated *IGHV* configuration (M‐CLL). Statistics were calculated using one‐way ANOVA followed by Tukey's post‐hoc test for three‐group comparison and Mann‐Whitney test for comparison between two groups. *** for *P* < .001, ** for *P* < .01 and * for *P* < .05; “n” refers to the number of patients. *GAPDH* was used as a housekeeping reference for qPCR analyses.

Given the established oncogenic function of PIM kinases in CLL cells,^9^, ^22^ we first assessed the expression of PIM1, PIM2 and PIM3 on both transcript and protein levels in a group of 88 newly diagnosed CLL patients, and the obtained results were correlated with the clinical data. In line with previous reports, CLL cells from our cohort expressed higher levels of all PIM kinase isoforms when compared to healthy B lymphocytes (Figure S1). Patients with advanced disease (Binet C) exhibited significantly higher PIM2 transcript and protein levels, and higher PIM1 protein expression than CLL patients at earlier stages (Binet A/B; Figure 1A, Figure S2A). Significantly higher PIM2 transcript/protein level and PIM1 protein expression were also observed at the time of diagnosis in patients, who progressed after first line treatment during follow‐up (median observation time = 27 months, range 2 to 230 months), (Figure 1B, Figure S2B). Moreover, patients with unmutated *IGHV* loci showed significantly higher PIM1 transcript level than patients with mutated *IGHV* genes (Figure 1C). In contrast, PIM3 transcript/protein abundance was not associated with any of clinical characteristics in CLL patients. These data indicate that PIM1 and PIM2 transcript and/or protein expression are increased in more aggressive CLL, prompting further questions about the mechanisms of their induction and consequences of their activity for the disease biology.

### 3.2 Microenvironment signals induce PIM expression

**Figure 2:**
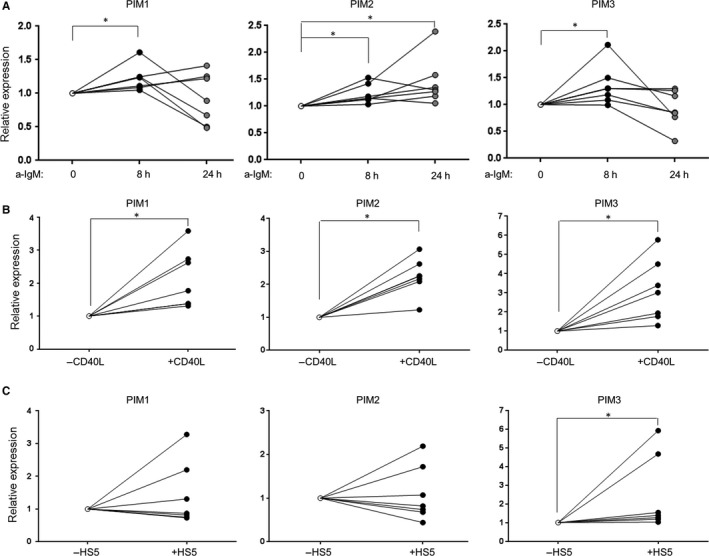
Microenvironment signals induce the expression of PIM kinases. CLL cells from 7 donors were incubated with 10 μg/mL anti‐IgM (a‐IgM) for 8 h and 24 h (A) or CD40L (50 ng/mL, 1 h) (B), or co‐cultured with HS5 cells for 48 h (C), and then collected for qPCR analyses. PIM1/2/3 transcript abundance was quantified using 2^−ΔΔ^ ^CT^ method, where *GAPDH* was used as a reference gene. The results are expressed relative to the value of untreated sample, assigned to an arbitrary value 1. * for *P* < .05; Wilcoxon matched pairs test.

Chronic lymphocytic leukaemia cell fate depends on microenvironment signals, which promote anti‐apoptotic and proliferative circuitry involving STAT and NFκB transcription factors.^6^, ^7^ Since these transcription factors are known PIM inducers,^23^ we hypothesized that expression of PIM kinases might be regulated by the interaction between CLL cells and their microenvironment. To test this hypothesis, we studied the ability of different microenvironmental stimuli to induce expression of PIM kinases. To mimic the B‐cell receptor activity, peripheral blood CLL cells were incubated with 10 μg/mL anti‐IgM for 8‐24 hours and then collected for quantitative PCR (qPCR) analysis. Activation of B‐cell receptor significantly increased the expression of all three PIM isoforms, leading to a sustained increase of PIM2 transcript levels and more transient up‐regulation of PIM1 and PIM3 (Figure 2A).

Contacts of CLL cells with T cells in the microenvironment engage pro‐survival, NFκB‐inducing CD40‐CD40L pathway.^24^ Thus, we assessed expression of PIM kinase isoforms after incubation of peripheral CLL cells with CD40 ligand (CD40L). Activation of the CD40 receptor in CLL cells significantly increased PIM1‐3 mRNA expression and protein abundance already after 1 hours of incubation (Figure 2B, Figure S3). Finally, we co‐cultured CLL cells with HS5 stromal cells. This interaction highly increased PIM3, but not PIM1 and PIM2 expression, when compared to CLL cells cultured without stromal support (Figure 2C). Taken together, these data indicate that PIM kinases are under the control of tumour microenvironment, but the pattern of response to external stimuli differs between PIM isoforms.

### 3.3 PIM inhibitor SEL24‐B489 induces apoptosis in CLL cells

**Figure 3:**
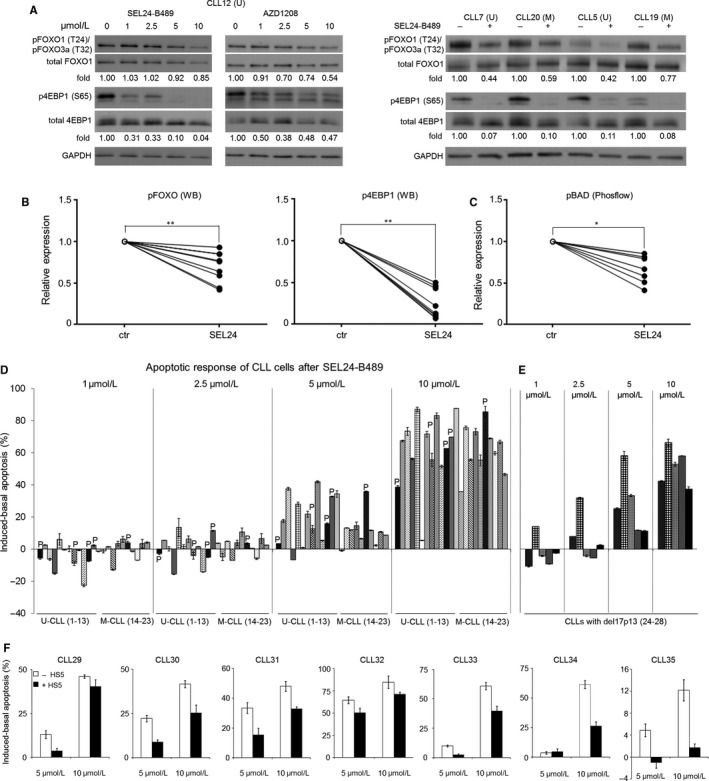
SEL24‐B489 decreases the phosphorylation of PIM substrates and induces CLL cell apoptosis. (A) Left: Pan‐PIM kinase inhibitors SEL24‐B489 and a referential compound, AZD1208, decrease the phosphorylation of PIM substrates pFOXO (T24/T32) and p4EBP1 (S65) in a dose dependent manner (1‐10 μmol/L, 24 h). Right: SEL24‐B489 (5 μmol/L, 24 h) decreases the levels of phosphorylated FOXO and 4EBP1 in both unmutated (U) and mutated (M) *IGHV* subtypes. Representative Western blots are shown. (B) Summary of changes in pFOXO (T24/T32) and p4EBP1 (S65) analysed in 8 patients by WB. Band intensities were quantified by densitometry. (C) SEL24‐B489 decreases phospho‐BAD levels in CLL cells. BAD serine 112 phosphorylation was assessed in 7 consecutive patients by intracellular phospho‐specific flow cytometry before and after incubation of CLL cells with SEL24‐B489 (5 μmol/L, 24 h). Statistics were calculated using Wilcoxon matched pairs test; ** and * indicate *P* < .01 and *P* < .05 respectively. (D) Apoptosis induction in 23 newly diagnosed CLL patients treated with increasing doses of SEL24‐B489 for 48 h. U‐CLL ‐ unmutated CLL, M‐CLL ‐ mutated CLL. “P” ‐ patients with disease progression. (E) Apoptotic response to SEL24‐B489 (1‐10 μmol/L, 48 h) in CLL cells with 17p13 deletion. (F) SEL24‐B489 overrides pro‐survival signals from HS5 cells. CLL cells co‐cultured with HS5 monolayers or grown alone were incubated with SEL24‐B489 (5 μmol/L and 10 μmol/L) for 48 h. The % of apoptotic cells was estimated by AnnexinV‐PE/7AAD staining. Bars represent mean ± SD from triplicates.

We next investigated the consequences of PIM inhibition in CLL cells using newly developed pan‐PIM inhibitor, SEL24‐B489.^20^, ^25^, ^26^ Incubation of CLL cells with 1‐10 μmol/L SEL24‐B489 for 24 hours caused a significant, dose‐dependent decrease in phosphorylation of PIM substrates: threonine 24/threonine 32 (T24/T32) of FOXO1/3a, serine 65 (S65) of 4EBP1 and serine 112 (S112) of BAD (Figure 3A‐C). SEL24‐B489 inhibitor decreased phosphorylation of these substrates irrespective of *IGHV* gene mutation status (Figure 3A, right panel). Similar effects were also observed with a referential pan‐PIM inhibitor, AZD1208, indicating that SEL24‐B489 induces expected, specific biochemical effects (Figure 3A, left panel).^27^ In contrast, SEL24‐B489 did not decrease the phosphorylation of FOXO1/3 and 4EBP1 in normal B lymphocytes (Figure S4A). Having confirmed inhibitor's on‐target activity, we next assessed the effect of SEL24‐B489 (1‐10 μmol/L, 48 hours) on viability of CD19+ CLL cells obtained from peripheral blood of 23 treatment‐naïve patients and 5 healthy individuals (Table S3). Incubation with SEL24‐B489 for 48 hours did not perturb normal B‐cells viability; in marked contrast, SEL24‐B489 triggered a dose‐dependent increase in apoptosis of CLL cells (Figure 3D, Figure S4B). Cells obtained from *IGHV*‐unmutated and *IGHV*‐mutated CLL patients were equally susceptible to SEL24‐B489‐induced apoptosis (Figure 3D, Table S3). CLL cells obtained from 5 patients who progressed after initial treatment also responded to the inhibitor, reaching 39%‐83% of apoptotic cells for a 10 μmol/L SEL24‐B489 dose (Figure 3D, Table S3). Importantly, CLL cells carrying del17p13/*TP53* point mutations were similarly sensitive to SEL24‐B489 as p53‐wild‐type cells (Figure 3D and E, Tables S1 and S3). Since microenvironment‐derived signals typically protect CLL cells from spontaneous and drug‐induced apoptosis, we examined sensitivity of CLL cells grown on HS5 monolayers to SEL24‐B489. In 6 out of 7 cases, the compound at least partially overrode the protective signals from HS5 cells and markedly triggered apoptosis (Figure 3F). It was previously shown that stromal cells induce CLL cells to express anti‐apoptotic MCL1 protein, which could account for HS5 chemoprotective effects.^28^ Thus, we assessed the effect of SEL24‐B489 on MCL1 expression in CLL cells co‐cultured with stromal cells. As expected, stromal cells increased MCL1 abundance in leukaemia cells. When SEL24‐B489 was added to co‐cultures, MCL1 expression in CLL cells markedly decreased (Figure S5A). Nonetheless, even in the presence of SEL24‐B489, stromal cells still afforded partial protection to CLL cells, suggesting involvement of other, PIM‐ and MCL1‐independent mechanisms in this protective cross‐talk (Figure S5B). Apoptosis induced by SEL24‐B489 in CLL cells incubated on HS5 monolayers was not linked to the *IGHV* mutation status.

### 3.4 SEL24‐B489 impairs CXCR4‐dependent migration

**Figure 4:**
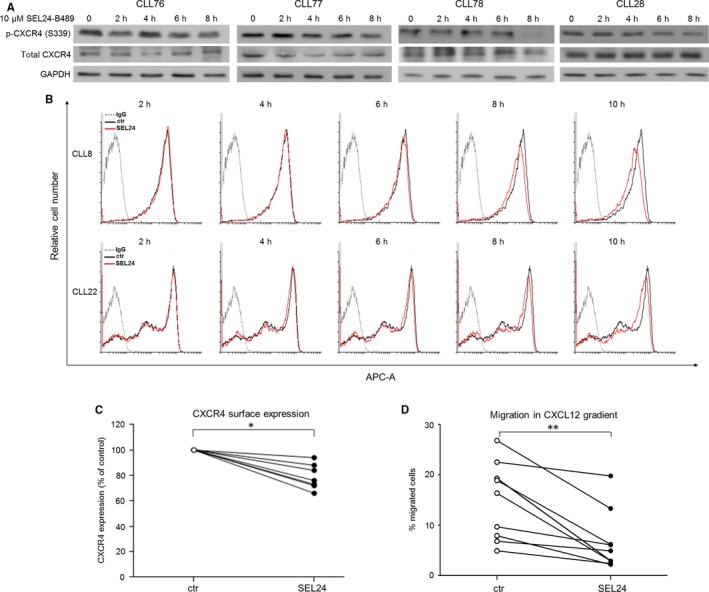
SEL24‐B489 impairs CXCR4‐mediated migration. (A) SEL24‐B489 causes time‐dependent decrease of p‐CXCR4 (S339) in CLL cells. (B) Example flow cytometry histograms of two CLL patients (CLL8, U‐CLL and CLL22, M‐CLL) showing time‐dependent decrease of CXCR4 surface expression after treatment with 10 μmol/L SEL24‐B489. Flow cytometry analysis was performed every 2 h after addition of the inhibitor. (C) SEL24‐B489 (10 μmol/L, 10 h) decreases the surface expression of CXCR4 (n = 7, *P < .05, Wilcoxon matched pairs test). (D) SEL24‐B489 reduces migration of CLL cells (n = 9) toward the CXCL12 ligand in a transwell assay (**P < .01, Wilcoxon matched pairs test).

PIM1 phosphorylates CXCR4 on serine 339 (S339) and increases CXCR4 surface expression and recycling.^8^, ^9^, ^25^ We thus explored whether pan‐PIM inhibitor SEL24‐B489 affects p‐CXCR4 levels and CXCR4‐driven migration in CLL cells. SEL24‐B489 caused time‐dependent reduction in p‐CXCR4 (S339) levels within 8 hours in all analysed samples (Figure 4A). These changes were followed by a decrease in CXCR4 surface expression, with the most noticeable effect observed after 10 hours (Figure 4B, C). Of note, 10 hours‐incubation with 10 μmol/L SEL24‐B489 did not induce apoptosis in CLL cells, as determined by AnnexinV‐PE/7AAD staining and analysis of PARP cleavage (Figure S6). To determine whether the decay in CXCR4 surface expression affects CXCR4‐dependent migration, CLL cells were treated with 10 μmol/L SEL24‐B489 or vehicle (DMSO) for 10 hours. After incubation, cell motility was assessed in a transwell assay. SEL24‐B489‐treated cells exhibited markedly reduced migration than control (DMSO‐treated) cells (Figure 4D).

### 3.5 PIMs modulate mTOR activity downstream of CXCR4

**Figure 5:**
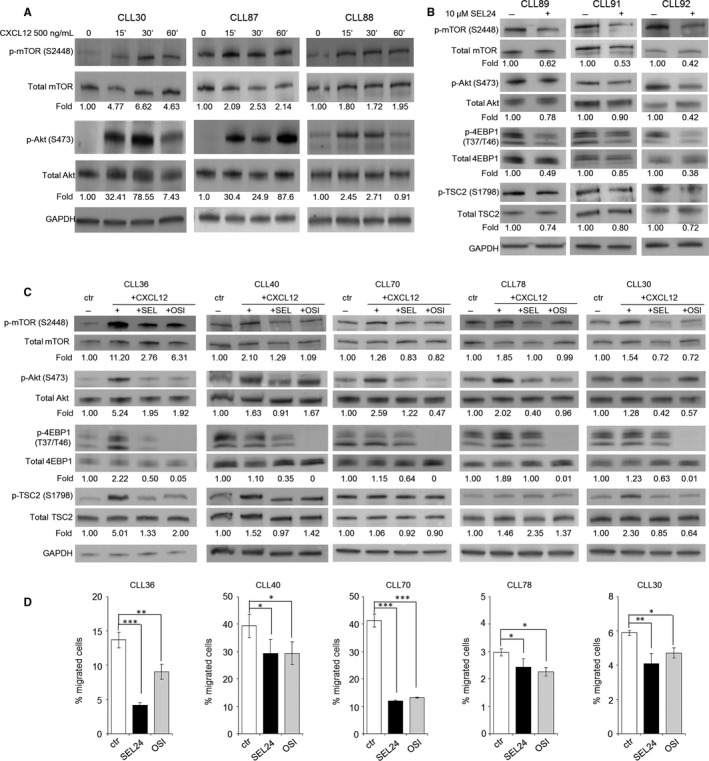
CXCR4/CXCL12 signal is transduced through mTOR pathway in a PIM‐dependent manner. (A) CXCL12 activates mTOR signalling pathway. After incubation with 500 ng/mL CXCL12 for 0‐60 min, primary CLL cells were lysed and assessed for p‐mTOR and p‐AKT levels by WB. Numbers below the blots indicate relative changes in phospho‐protein abundance, determined by densitometric quantification using Image Studio Lite programme. (B) SEL24‐B489 blocks the baseline activity of mTOR pathway. Primary CLL cells were incubated with 10 μmol/L SEL24‐B489 for 1 h. Changes in phospho‐protein abundance were determined by Western blotting, and quantified using Image Studio Lite programme. (C) Pan‐PIM inhibitor SEL24‐B489 and mTOR inhibitor OSI‐027 inhibit CXCL12‐activated mTOR pathway. CLL cells were pre‐incubated with 10 μmol/L SEL24‐B489 or OSI‐027 for 1 h and then stimulated with CXCL12 (500 ng/mL, 15 min). (D) Inhibition of PIM and mTOR kinases impairs CLL cells migration in the CXCL12 gradient. Primary CLL cells were pretreated with SEL24‐B489 or OSI‐027 (both at 10 μmol/L) and placed in a transwell chamber in CXCL12 gradient. Numbers of migrated cells were determined after 6 h using trypan blue exclusion assay. Bars represent mean ± SD from triplicates, ***P < .001, **P < .01 and *P < .05 calculated with Mann–Whitney test.

Given the marked decrease in CXCR4‐driven migration caused by PIM inhibition and its relatively moderate effect on CXCR4 surface expression, we hypothesized that PIM kinases influence CXCR4‐driven migration by an additional mechanism. We found that incubation of leukaemia cells with CXCL12 led to increased phosphorylation of mTOR (S2448) and AKT (S473), revealing CXCL12‐mediated activation of this pathway in CLL (Figure 5A). To determine whether PIM inhibition interferes with mTOR pathway, we first incubated CLL cells with SEL24‐B489 and found decreased phosphorylation of mTOR pathway components, including p‐mTOR (S2448), p‐TSC2 (S1798) and p‐AKT (S473; Figure 5B). Consistent with this, we also found decreased phosphorylation of direct mTOR substrates, 4EBP1 serine 37 and threonine 46 (S37/T46), indicating that PIM inhibition blocks signalling through mTOR pathway (Figure 5B).^29^ We next determined whether PIM inhibition could block CXCL12‐mediated mTOR activation. As expected, SEL24‐B489 or OSI‐027 (an mTORC1/2 inhibitor) markedly decreased CXCL12‐induced phosphorylation of mTOR pathway components, demonstrating that PIM inhibitors block CXCR4‐dependent mTOR activation and signalling (Figure 5C). We next investigated the impact of mTOR inhibition on CXCR4‐dependent migration. For these experiments, CLL cells were incubated with 10 μmol/L SEL24‐B489 or 10 μmol/L mTOR inhibitor OSI‐027 for 10 hours, then the inhibitors were washed out and cells were proceeded to migration assays. Of note, under these conditions, neither SEL24‐B489 nor OSI‐027 decreased cell viability, as assessed by AnnexinV‐PE/7‐AAD double staining and analysis of PARP cleavage (Figure S6). As shown in Figure 5D, pre‐incubation of primary CLL cells with mTOR inhibitor OSI‐027 impaired CLL cells migration towards CXCL12 gradient to a similar degree as SEL24‐B489. Taken together, these findings indicate that CXCR4‐triggered mTOR pathway is essential for CLL migration in CXCL12 gradient. Furthermore, PIM inhibition decreases CLL cell chemotaxis in a mechanism that involves decreased CXCR4 surface expression and inhibition of CXCR4‐induced mTOR pathway.

## 4 DISCUSSION

Oncogenic PIM kinases are frequently overexpressed by human lymphoid malignancies, supporting antiapoptotic signalling and cellular proliferation. Expression of PIMs is also a common feature of CLL cells, where they play similar pro‐survival functions.^9^, ^22^, ^30^ Unlike in the case of many other oncogenic kinases, PIMs’ deregulated activity in tumours is not due to gene fusions or activating mutations. Instead, PIM kinases are usually induced by the transcription factors acting downstream of growth factors and cytokines, such as the STATs and NFκB.^23^, ^31^, ^32^ As PIMs do not require post‐translational modifications for activity—once expressed, they constitutively exhibit oncogenic function.^33^ CLL lymph node microenvironment is an ample source of signals triggering NFκB pathway, for example, BCR signalling or ligation of TNF family receptors, such as CD40.^34^, ^35^ Of note, both these receptors lead also to a delayed induction of STATs activity, cooperating with NFκB in PIMs transcriptional regulation.^36^, ^37^ Consistent with this, *in vitro* stimulation of CLL cells with CD40 ligands or BCR crosslinking led to induction of PIM expression. In addition, PIM1 gene expression was markedly higher in unmutated *IGVH* CLL cells, which typically exhibit higher levels of BCR activity. In line with the role of BCR signal in PIM1 regulation, a BTK inhibitor, ibrutinib, decreased expression of PIM1 in murine TCL1‐192 cells xenografted to SCID mice.^8^ Taken together, these observations indicate that induction of PIM kinases in CLL is controlled by microenvironment signals.

Given the pleiotropic pro‐survival functions of PIMs, their increased expression in the lymph node‐resident CLL cells will likely facilitate drug resistance, increase metabolic fitness and protect CLL cells from proapoptotic stimuli.^38^, ^39^ Accordingly, inhibition of PIMs in CLL cells with SEL24‐B489 decreased phosphorylation of PIM substrates (p‐FOXO1, p‐4EBP1 and p‐BAD) and exhibited direct proapoptotic activity, regardless of the support from stromal cells. Importantly, the PIM inhibitor did not affect the viability of normal B lymphocytes and was equally effective in triggering apoptosis in CLL cells from patients with 17p/*TP53* abnormalities as in *TP53* wild‐type cells. These observations are particularly important, as genetic abnormalities and/or indirect inactivation of the *TP53* tumour suppressor pathway are the most significant predictors of poor outcome after standard chemotherapy.^2^, ^19^ Thus, from the clinical standpoint, therapeutic interventions capable of inducing apoptosis in p53‐independent fashion are particularly desired for CLL patients. In fact, since PIM kinases directly modulate the activity and expression of multiple BCL2‐family members, their inhibition might trigger the mitochondrial cell death pathway and cytochrome C release independently of p53. For example, PIMs regulate BAD phosphorylation on the S112 gatekeeper site, restoring its pro‐apoptotic activity.^40^ In addition, inhibition of PIM‐dependent protein translation decreases abundance of an antiapoptotic BCL2 family protein, MCL1.^30^ We show here that PIM kinase inhibition also markedly reduced the expression of MCL1 protein induced by stromal cell contact. Thus, PIM inhibition triggers proapoptotic mechanisms that are not blocked, or only partially blocked, by microenvironmental support, resulting in p53‐independent cell death.^41^

The homing of CLL cells to a proliferation‐conducive and protective lymphoid compartments is predominantly regulated by CXCR4 chemokine receptor.^6^ Thus, interference with the activation of CXCR4 receptor in CLL cells facilitates their egress from the lymph node niche and/or prevents their homing to lymphoid organs.^9^ As a consequence, CLL cells re‐localized from the lymph nodes to peripheral blood are deprived of the microenvironment signals, cease to proliferate and are more susceptible to drug‐induced apoptosis. PIM kinases (predominantly PIM1) are the key regulators of CXCR4 (S339) phosphorylation, facilitating membrane retention of the receptor in myeloid and lymphoid cells.^9^, ^42^ Inhibition of PIMs with the newly developed SEL24‐B489 pan‐PIM inhibitor in primary CLL cells led to a time‐dependent decrease of S339 phosphorylation and concurrent decrease in CXCR4 surface membrane expression. Similar reductions in CXCR4 surface expression in CLL cells were previously noted with a different PIM inhibitor, K00135.^9^ In addition, PIM inhibitor in this study also blocked re‐externalization of the CXCR4 receptor, internalized after CXCL12 ligation.

Decreased expression of surface CXCR4 after incubation with SEL24‐B489 resulted eventually in diminished migration of CLL cells toward the CXCL12 gradient. Our further studies revealed that activation of CXCR4 by its ligand induces mTOR signalling, and mTOR inhibitors abrogate CXCL12 mediated chemotaxis. Thus, these data indicate that mTOR pathway downstream of CXCR4 is essential for directional migration of CLL cells. As PIM kinases regulate the mTOR signalling at least *via* two independent pathways, involving PIM2‐mediated phosphorylation of TSC2 (S1798) and PIM1‐mediated phosphorylation of PRAS40 (T240), PIM inhibition attenuates mTOR activity and decreases CXCR4/mTOR‐mediated chemotaxis.^43^, ^44^ Collectively, these data indicate that PIMs play an important role in CLL cell life cycle, linking microenvironment with CXCR4‐mediated migration and pro‐survival signalling. In the lymph node, microenvironment‐induced PIM kinases facilitate tethering of CLL cells to stroma and promote pro‐survival signalling. PIM inhibitor not only directly targets CLL cells, but also abrogates the cross‐talk between leukaemic cells and their microenvironment by impairing CXCR4‐CXCL12 interaction and mTOR activity, leading to detachment of CLL cell from the niche and facilitating apoptosis.

Consistent with hitherto identified pathogenetic functions of PIM kinases, expression of individual PIM isoforms in CLL patients was associated with disease aggressiveness and outcome. We demonstrated that higher PIM1 and PIM2 transcript and/or protein abundance was characteristic for subjects with more advanced disease at diagnosis, and those who relapsed after first‐line treatment. Since PIM kinase isoforms are transcriptionally regulated by STAT and NFκB transcription factors, and both STAT and NFκB expression in CLL cells are associated with inferior prognosis, these proteins are likely inducers of PIMs in more advanced or chemoresistant CLL.^45^ Transcriptionally up‐regulated PIMs further potentiate disease aggressiveness *via* multiple mechanisms promoting tumour cell survival and growth.

These observations link expression of PIM kinases with CLL clinical behaviour, underscore their role of tumour biology modifiers and rationalize further clinical development of PIM kinase inhibitors in CLL. Although PIM isoforms are highly homologous and share an overlapping spectrum of substrates, in CLL cells they exhibit non‐overlapping, yet highly complementary functions, whereby PIM1 is essential for CXCR4‐dependent CLL cell migration and PIM2/3 for cell survival.^9^, ^15^, ^46^ Thus, inhibition of all PIM isoforms is a rational approach, excluding possibility of isoform redundancy or compensation issues. In this study, we utilized a newly developed, small molecule pan‐PIM inhibitor, SEL24‐B489, which previously showed favourable toxicity profile and encouraging results in preclinical AML models.^25^ SEL24‐B489 is currently under clinical development in a phase I/II trial in AML patients (clinicaltrials.gov: NCT03008187). Taken together, our results suggest that SEL24‐B489 pan‐PIM inhibitor may be a new valuable approach toward CLL therapy. Further studies are warranted to test this opportunity.

## CONFLICT OF INTEREST

P. Juszczyński is a member of the Scientific Advisory Board at Selvita S.A. and served as a consultant for Selvita S.A. Dr. Czardybon, Dr. Gałęzowski, Dr. Windak and Dr. Brzózka are Selvita S.A. employees.

## AUTHORS CONTRIBUTIONS

EB designed and performed most of the experiments, analysed data and wrote the paper. PG performed the experiments and analysed data. MNK and KP performed experiments and isolated leukaemic cells. HML and GN performed *IGHV* mutational status analyses. AB and KB performed cytogenetic analyses. MS, EJ, TS, and AP performed experiments. BP, ELM, BKB, MWJ and KW collected and analysed the clinical data. WC, MG, RW and KBr performed statistical analyses and wrote the paper. PJ designed the research study and wrote the paper.
