# Supplementary material for: Microenvironment‐induced PIM kinases promote CXCR4‐triggered mTOR pathway required for chronic lymphocytic leukaemia cell migration
Source: J Cell Mol Med. 2018 Apr 17;22(7):3548–59. doi: 10.1111/jcmm.13632 (PMC6010703; doi:10.1111/jcmm.13632)

**Supplementary Figures and Information**

**Microenvironment-induced PIM kinases promote CXCR4-triggered mTOR pathway required for chronic lymphocytic leukemia cell migration**

Emilia Bialopiotrowicz^1^, Patryk Gorniak^1^, Monika Noyszewska-Kania^1^, Bartosz Pula^2^, Hanna Makuch-Lasica^3^, Grazyna Nowak^3^, Aleksandra Bluszcz^3^, Maciej Szydlowski^1^, Ewa Jablonska^1^, Karolina Piechna^1^, Tomasz Sewastianik^1^, Anna Polak^1^, Ewa Lech-Maranda^2,4^, Bozena K. Budziszewska^2,4^, Maja Wasylecka-Juszczynska^1^, Katarzyna Borg^3^, Krzysztof Warzocha^2^, Wojciech Czardybon^5^, Michal Galezowski^5^, Renata Windak^5^, Krzysztof Brzozka^5^ and Przemyslaw Juszczynski^1^ **^*^**

^1^Department of Experimental Hematology, Institute of Hematology and Transfusion Medicine, Warsaw, Poland

^2^Department of Hematology, Institute of Hematology and Transfusion Medicine, Warsaw, Poland

^3^Department of Diagnostic Hematology, Institute of Hematology and Transfusion Medicine, Warsaw, Poland

^4^Department of Hematology and Transfusion Medicine, Centre of Postgraduate Medical Education, Warsaw, Poland

^5^Selvita S.A., Cracow, Poland

**(*) the address of the author for correspondence:** Prof. Przemyslaw Juszczynski, address: Institute of Hematology and Transfusion Medicine, Department of Experimental Hematology, I. Gandhi 14 Str, Warsaw, 02-776, Poland; e-mail: pjuszczynski@ihit.waw.pl, tel.: +48 22 3496 477; fax: +48 22 3496 237

**Supplemental Table SI. Primers used for qPCR**

| **primer** | **sequence** |
| --- | --- |
| PIM1_F | 5’-ACTGGGGAGAGCTGCCTAATG-3’ |
| PIM1_R | 5’-AGGATCAGGACGAAACTGTCG-3’ |
| PIM2_F | 5’-CATCGTGACATCAAGGATGAGAA-3’ |
| PIM2_R | 5’-CCTTGTCCCATCAAAGTCAGTGT-3’ |
| PIM3_F | 5'- TCTCTCCAGAGTGCCAGCA -3' |
| PIM3_R | 5'- GTGCACAGCCGCAGGTCA-3' |
| GAPDH_F | 5’-AGCCTCCCGCTTCGCTCTCT-3’ |
| GAPDH_R | 5’-CGACCAAATCCGTTGACTCCGAC-3’ |

**Supplemental Table SII. Antibodies used in the study**

| **antibody** | **origin** | **company** | **Catalog number** |
| --- | --- | --- | --- |
| PIM1 | rabbit | Abcam | 75776 |
| PIM2 (D1D2) | rabbit | Cell Signaling Technology | 4730 |
| PIM3 (D17C9) | rabbit | Cell Signaling Technology | 4165 |
| Bcl-xL (54H6) | rabbit | Cell Signaling Technology | 2764 |
| p-FOXO1(T24)FOXO3(T32) (4G6) | rabbit | Cell Signaling Technology | 2599 |
| FOXO1 (C29H4) | rabbit | Cell Signaling Technology | 2880 |
| p-4EBP1(S65) (174A9) | rabbit | Cell Signaling Technology | 9456 |
| p-4EBP1(T37/T46) (236B4) | rabbit | Cell Signaling Technology | 2855 |
| 4EBP1 (53H11) | rabbit | Cell Signaling Technology | 9644 |
| Mcl1 (D35A5) | rabbit | Cell Signaling Technology | 5453 |
| PARP (46D11) | rabbit | Cell Signaling Technology | 9532 |
| p-mTOR(S2448) | rabbit | Cell Signaling Technology | 2971 |
| mTOR | rabbit | Cell Signaling Technology | 2972 |
| p-Akt (S473) (D9E) | rabbit | Cell Signaling Technology | 4060 |
| Akt (11E7) | rabbit | Cell Signaling Technology | 4685 |
| p-TSC2 (325.S1798) | mouse | Santa Cruz Biotechnology | sc-293149 |
| TSC2 (28A7) | rabbit | Cell Signaling Technology | 3635 |
| p-CXCR4(S339) | rabbit | Abcam | ab74012 |
| CXCR4 | goat | Abcam | ab1670 |
| p-BAD (S112) | rabbit | Cell Signaling Technology | 9291 |
| BAD | rabbit | Cell Signaling Technology | 9292 |
| DA1E mAb IgG XP isotype control | rabbit | Cell Signaling Technology | 3900 |
| GAPDH (6C5) | mouse | Merck Millipore | MAB374 |
| Anti-rabbit IgG (whole molecule)-peroxidase | goat | Sigma-Aldrich | A0545 |
| Anti-goat IgG (whole molecule)-peroxidase | rabbit | Sigma-Aldrich | A5420 |
| Anti-mouse IgG (whole molecule)-peroxidase | rabbit | Sigma-Aldrich | A9044 |
| FITC anti-human CD19 (HIB19) | mouse | BD Pharmingen | 555412 |
| APC anti-human CD184 (CXCR4) (12G5) | mouse | BioLegend | 306509 |
| Anti-rabbit IgG (H+L) Fab2 Alexa Fluor 488 | goat | Cell Signaling Technology | 4412 |
| APC mouse IgG2a, κ isotype ctrl | mouse | BioLegend | 400219 |

**Supplemental Figure S1. Expression of PIM kinases in CLL cells compared to cells from healthy individuals (ctr1-ctr3).** Peripheral CD19+ cells were obtained from CLL patients and healthy volunteers using immunomagnetic selection, lysed and immunoblotted with indicated antibodies.


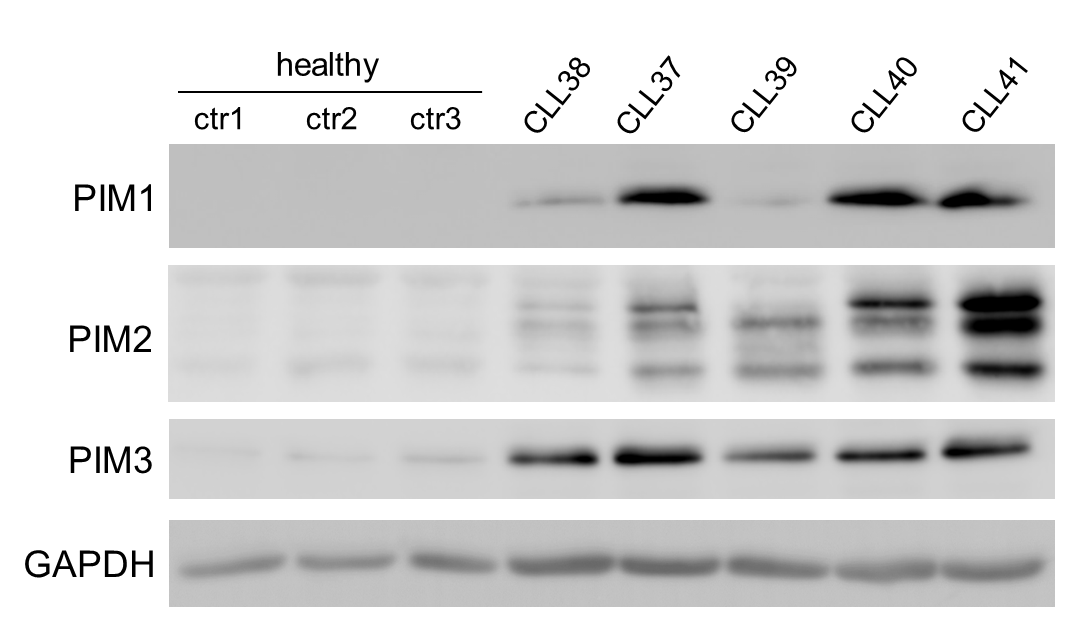


**Supplemental Figure S2. PIM expression is associated with CLL clinical parameters.** PIM1/2 protein expression in CLL patients was determined by quantitation of western blots using Image Studio Lite program. Example source western blots are shown. **(A)** PIM1 and PIM2 protein expression in patients with advanced CLL (Binet C) compared to subjects in earlier disease stages (Binet A/B). **(B)** PIM1 and PIM2 protein expression at diagnosis in patients who progressed after first line treatment during follow up versus those who remained in remission.

**
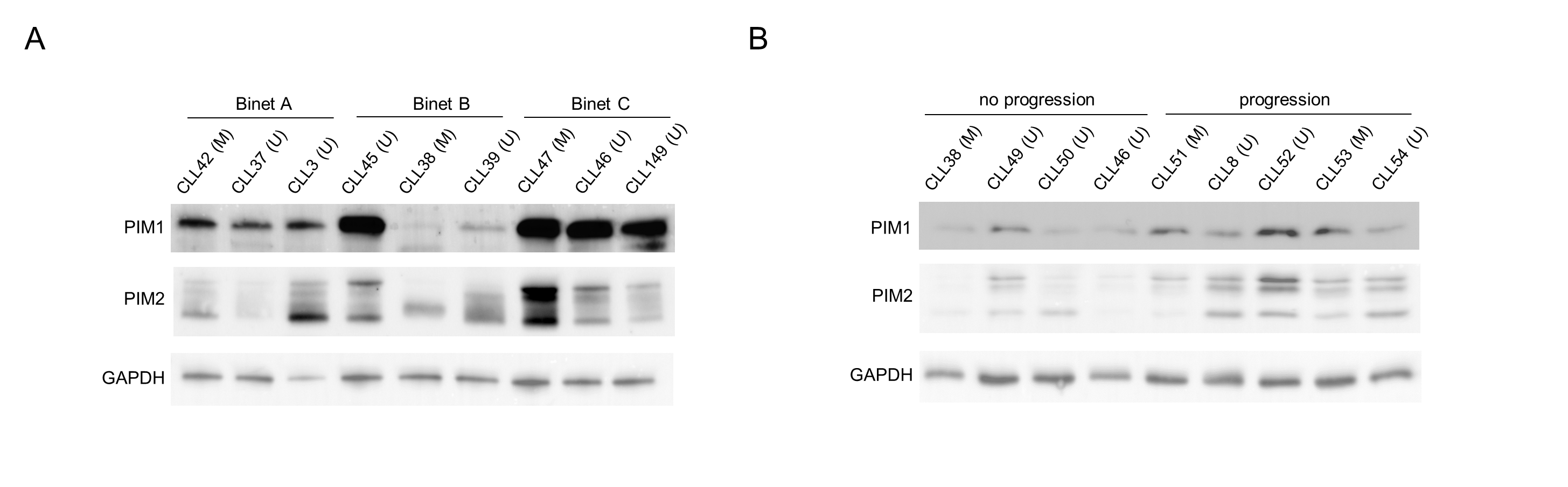
**

**Supplemental Figure S3. Induction of PIMs protein expression after incubation with CD40 ligand (CD40L).** CLL cells from 2 representative patients (CLL64 & CLL65) were incubated with 50ng/ml CD40L for 1 or 4 hours, collected, and analyzed for PIMs protein expression using western blotting. CD40-inducible Bcl-xL protein expression was analyzed as a positive control. Proteins levels were quantified and normalized to GAPDH. Protein abundance in vehicle-treated cells was arbitrarily assigned as 1. Indicated expression values are calculated relatively to the vehicle-treated cells.


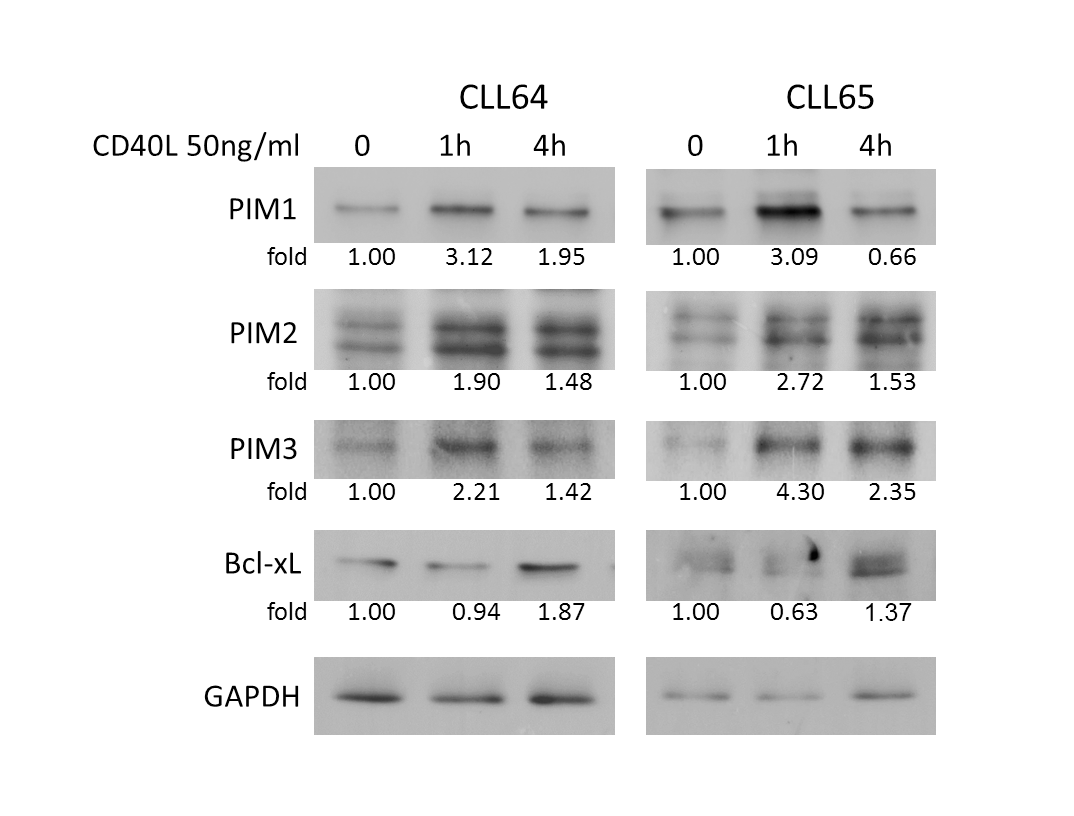


**Supplemental Figure S4. Effect of SEL24-B489 on PIMs substrate phosphorylation and apoptotic response in healthy B lymphocytes.** (**A**) Analysis of phosphorylated PIMs substrates after incubation with SEL24-B489 (5µM, 24h) in B lymphocytes derived from healthy donors (ctr1-ctr3). Densitometric analysis was performed using Image Studio Lite program; GAPDH served as a loading control. The phosphorylation level of PIMs substrates after SEL24-B489 was expressed relatively to DMSO-treated cells (assigned as a value of 1). (**B**) Peripheral CD19+ B-cells obtained from healthy volunteers (ctr1-ctr5) were incubated with SEL24-B489 (0-10μM) for 48h. Thereafter cells were stained with AnnexinV-PE/7-AAD and analyzed using flow cytometry. For comparison, example CLL patient (CLL7) is shown.

**
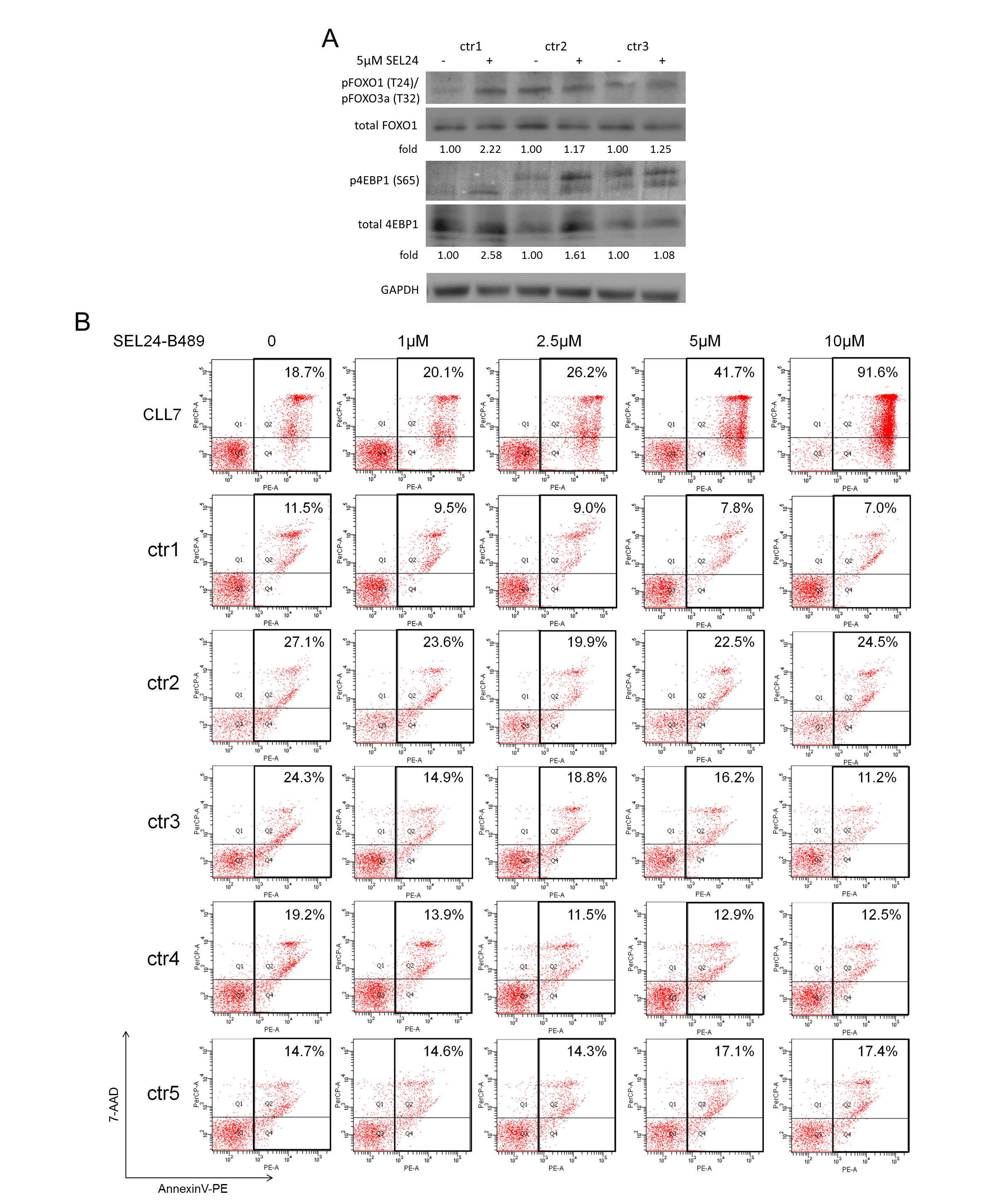
**

**Supplemental Figure S5. PIM inhibition decreases stromal cell contact-induced MCL1 protein expression in CLL cells. (A)** CLL cells were cultured on HS5 cell monolayers in the presence of vehicle (DMSO) or SEL24-B489 (5-10 μM). MCL1 expression in CLL cells was assessed by Western blotting. Four representative CLL patients are shown. Densitometric analysis was referred to GAPDH and normalized to MCL1 level of CLL cells grown without HS5 support (assumed as arbitrary value 1). **(B)**  SEL24-B489 partially overrides the protective effects afforded by stromal cells. * for p<0.05 calculated with Wilcoxon matched pairs test

.


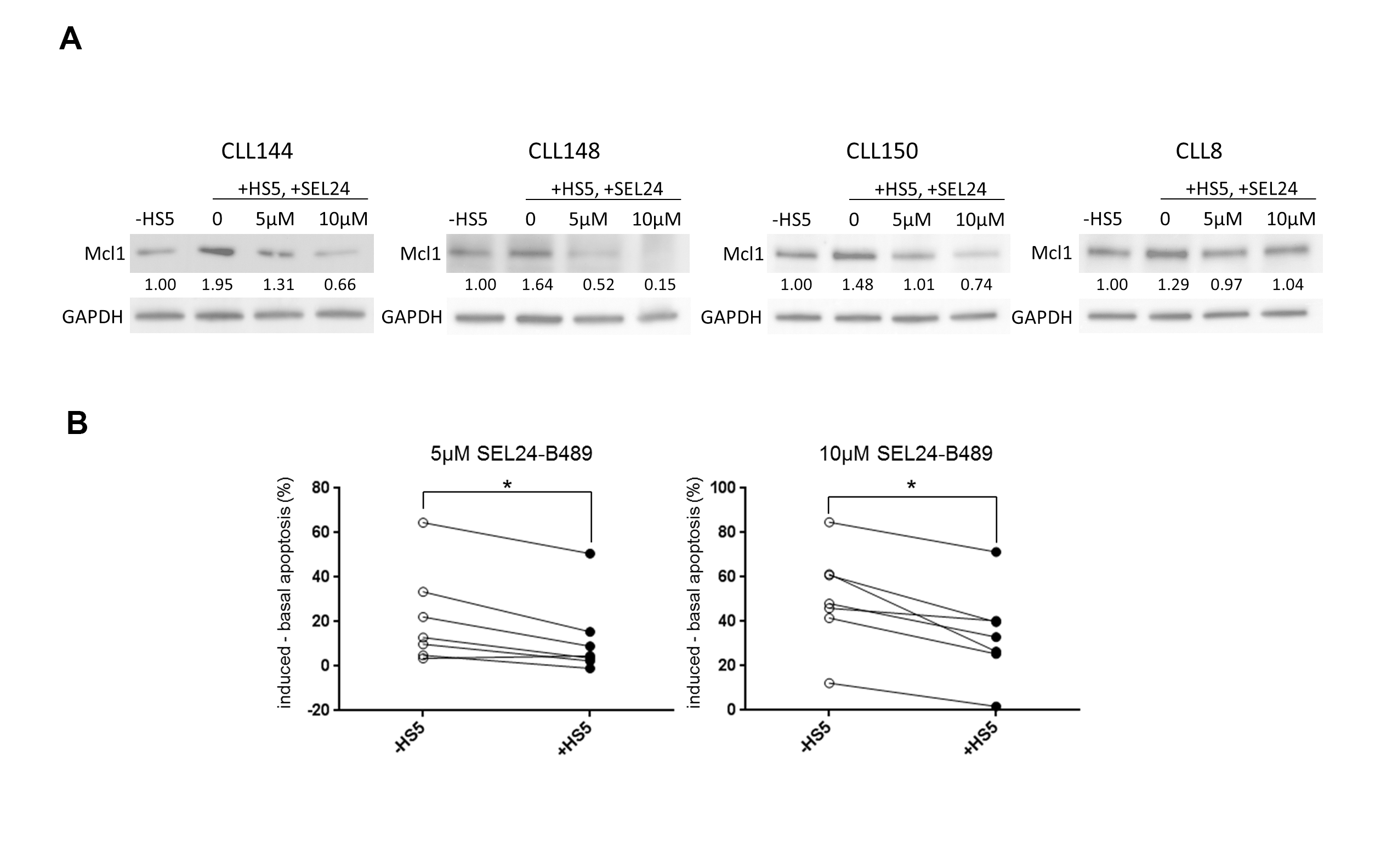


**Supplemental Table SIII. Characteristics of CLL patients studied for apoptotic response to SEL24-B489**

| **patient** | **sex** | ***IGHV* status** | **Binet** | **progression/**  **(1st line treatment)** | **del13q14 (*mir-15a* and**  ***mir-16-1*)** | **del17p13**  **(*TP53*)**  **or *TP53* gene mutation** | **del11q23**  **(*ATM*)** | **centromere**  **12** |
| --- | --- | --- | --- | --- | --- | --- | --- | --- |
| CLL1 | F | U-CLL | C | + /(6 x R-CHOP) | polyploidy | - | polyploidy | polyploidy |
| CLL2 | M | U-CLL | A | - | hom 81% | - | hom 75% | - |
| CLL3 | M | U-CLL | B | - | - | - | hom 10% | - |
| CLL4 | M | U-CLL | A | - | hom 89% | - | - | - |
| CLL5 | M | U-CLL | A | - | hom 93% | - | - | - |
| CLL6 | F | U-CLL | A | - | hom 88% | - | - | - |
| CLL7 | M | U-CLL | C | - | hom 95% | - | - | - |
| CLL8 | F | U-CLL | B | + /(3 x R-FC) | hom 99% | - | - | - |
| CLL9 | F | U-CLL | B | - | hom 42% | - | - | - |
| CLL10 | M | U-CLL | C | - | hom 90% | - | hom 93% | - |
| CLL11 | M | U-CLL | C | + /(leukapheresis + 5 x R-FC) | hom 17% | - | - | - |
| CLL12 | M | U-CLL | B | + /(5 x leukeran) | hom 40%  het 55% | - | - | - |
| CLL13 | F | U-CLL | B | - | - | 13359C>CT:241S>F | - | - |
| CLL14 | M | M-CLL | B | - | hom 68% | - | - | - |
| CLL15 | F | M-CLL | A | - | - | - | - | - |
| CLL16 | M | M-CLL | B | - | - | - | hom 91% | - |
| CLL17 | M | M-CLL | A | - | hom 87% | - | - | - |
| CLL18 | F | M-CLL | C | - | - | - | - | - |
| CLL19 | F | M-CLL | A | +/(2 x 2-CDA) | - | - | - | - |
| CLL20 | F | M-CLL | B | - | hom 53%,  het 32% | - | - | - |
| CLL21 | M | M-CLL | A | - | - | - | - | - |
| CLL22 | M | M-CLL | B | - | hom 18%,  het 63% | - | - | - |
| CLL23 | M | M-CLL | A | - | hom 85%  het 10% | - | - | - |

M - male; F - female; U-CLL - patients with unmutated *IGHV* genes; M-CLL - patients with mutated *IGHV* genes,
R-CHOP=rituximab+cyclophosphamide+vincristine+prednisone+doxorubicin; FC= cyclophosphamide+fludarabine;

R-FC=rituximab+cyclophosphamide+fludarabine; 2-CDA=cladribine; “%” refers to the percentage of cells with homozygotic (hom) or heterozygotic (het) deletion.

**Supplemental Table SIV. Characteristics of CLL patients with del17p13 studied for apoptotic response to SEL24-B489**

| **patient** | **sex** | **Binet** | **progression/(treatment scheme)** | **del13q14 (*mir-15a* and**  ***mir-16-1*)** | **del17p13**  **(*TP53*)** | **del11q23**  **(*ATM*)** | **centromere 12** |
| --- | --- | --- | --- | --- | --- | --- | --- |
| CLL24 | M | C | +/(1 x CC; 6 x R-Solu-Medrol; 1 x CC) | - | hom 33% | - | - |
| CLL25 | F | C | +/(8 x leukeran; 5 x 2-CDA; 3 x 2-CDA; 4 x R-FC; 3 x FC; 6 x R-Solu-Medrol; 2 x Benda-Dex) | - | hom 20% | - | - |
| CLL26 | M | B | +/ (6 x R-CC + 2 x Mabthera; 6 x R-B) | hom 6%  het 89% | hom 92% | - | - |
| CLL27 | F | B | +/5x B; 7x Solu-Medrol; 6x R-CHOP; ibrutinib | hom 91% | hom 14% | - | - |
| CLL28 | M | B | newly diagnosed (before treatment) | hom 88% | hom 70% | - | - |

M - male; F- female; CC=cladribine+cyclophosphamide ; 2-CDA=cladribine; R-CC=rituximab+ cladribine+cyclophosphamide; B=bendamustine; R-B=rituximab+bendamustine; R-CHOP=rituximab+ cyclophosphamide+vincristine+prednisone+doxorubicin; FC= cyclophosphamide+fludarabine;
R-FC=rituximab+cyclophosphamide+fludarabine; “%” refers to the percentage of cells with homozygotic (hom) or heterozygotic (het) deletion.

**Supplemental Figure S6. Effect of 10µM SEL24-B489 and 10µM OSI-027 on CLL cells viability in time.** (**A**) CLL cells viability after incubation with 10µM SEL24-B489 (left) or 10µM OSI-027 (right) was assessed using Annexin V-PE/7AAD staining. Double negative cells were considered live and referred to the DMSO-treated control, which was assumed as 100% viability. Graphs represent mean ± SD for 5 and 3 example patients for SEL24-B489 and OSI-027, respectively. (**B**) Representative western blots showing PARP cleavage in a representative CLL sample incubated with 10µM SEL24-B489 (left) or 10µM OSI-027 (right) for 4-48h.


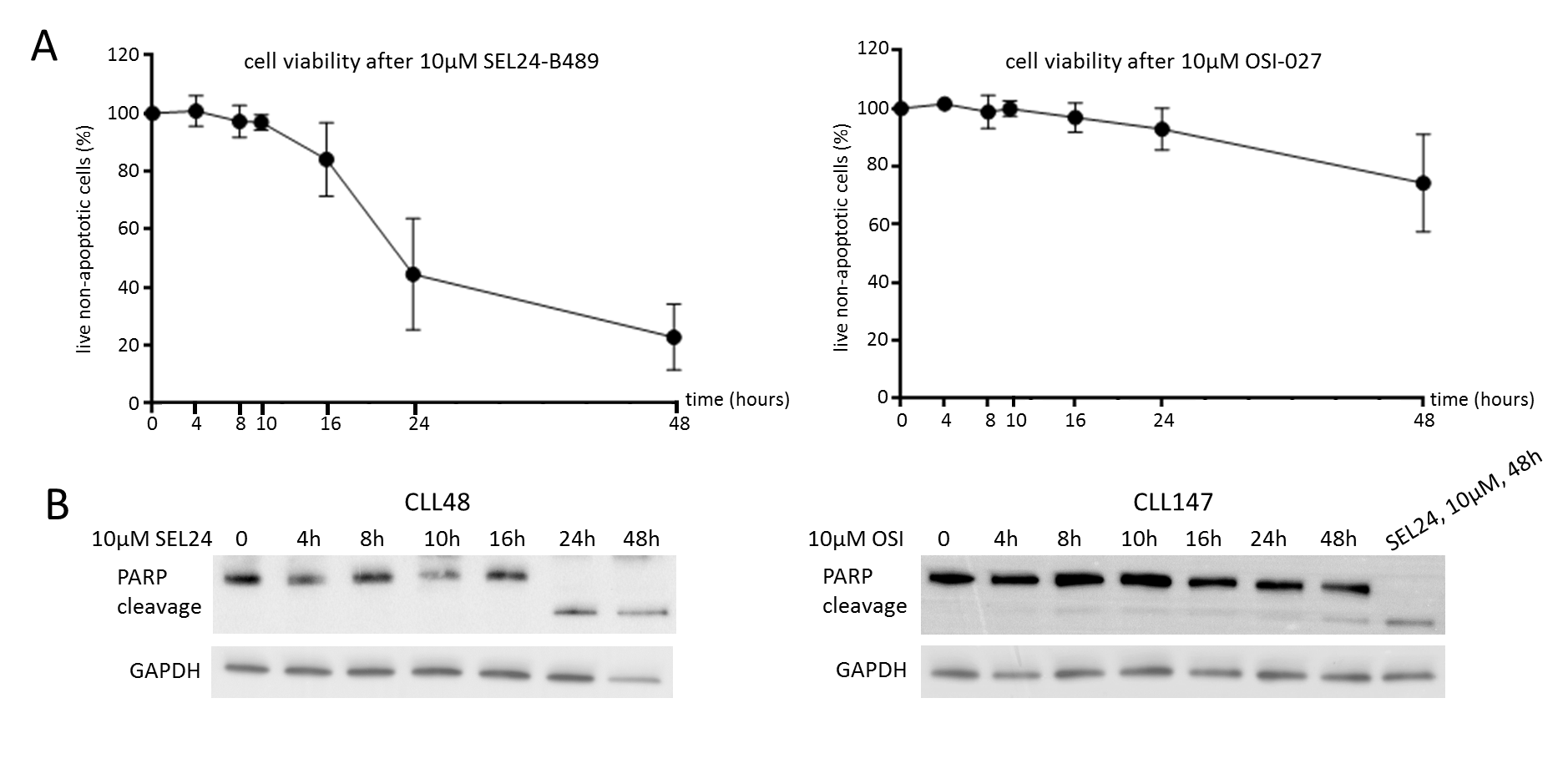

Supplement: Supplementary file 1 [file JCMM-22-3548-s001.doc]
